# Maternal Supplementation with Polyphenols and Omega-3 Fatty Acids during Pregnancy: Effects on Growth, Metabolism, and Body Composition of the Offspring

**DOI:** 10.3390/ani10111946

**Published:** 2020-10-22

**Authors:** Ana Heras-Molina, José Luis Pesantez-Pacheco, Susana Astiz, Consolación Garcia-Contreras, Marta Vazquez-Gomez, Teresa Encinas, Cristina Óvilo, Beatriz Isabel, Antonio Gonzalez-Bulnes

**Affiliations:** 1 SGIT-INIA, Ctra. De La Coruña Km. 7.5, 28040 Madrid, Spain; delasheras.ana@inia.es (A.H.-M.); jose.pesantez@ucuenca.edu.ec (J.L.P.-P.); astiz.susana@inia.es (S.A.); congarcon@gmail.com (C.G.-C.); ovilo@inia.es (C.Ó.); 2 School of Veterinary Medicine and Zootechnics, Faculty of Agricultural Sciences, University of Cuenca, Avda. Doce de Octubre, Cuenca 010220, Ecuador; 3 Faculty of Veterinary Medicine, UCM, Ciudad Universitaria s/n, 28040 Madrid, Spain; martavazgomez@gmail.com (M.V.-G.); tencinas@vet.ucm.es (T.E.); bisabelr@ucm.es (B.I.); 4 Facultat de Veterinària, Universitat Autònoma de Barcelona, Edifici V, Trav. dels Turons, 08193 Bellaterra, Spain

**Keywords:** antioxidants, fatty-acids, intrauterine-growth-restriction, pregnancy, swine-model

## Abstract

**Simple Summary:**

The present study aimed to determine benefits and risks of a dietary supplementation combining hydroxytyrosol and n3 polyunsaturated fatty acids (PUFA) on developmental patterns and metabolic traits of offspring in swine, a model of intrauterine growth restricted (IUGR) pregnancies. There were no deleterious effects on the reproductive traits of the sows and the postnatal features of the piglets. Piglets from the supplemented sows, in spite of a lower mean weight and corpulence at birth, showed higher average daily weight gain and fractional growth rate afterwards. Consequently, they reached higher weight and corpulence with increased muscle development and better lipidemic and fatty acids profiles than control offspring at juvenile stages.

**Abstract:**

Maternal supplementation with antioxidants and n3 PUFAs may be a promising strategy to reduce the risk of intrauterine growth restriction and preterm delivery, which may diminish the appearance of low-birth-weight neonates. The present study aimed to determine benefits and risks of a dietary supplementation combining hydroxytyrosol, a polyphenol from olive leaves and fruits, and n3 PUFAs, from linseed oil, on developmental patterns and metabolic traits of offspring in swine, a model of IUGR pregnancies. The results obtained indicate that maternal supplementation with hydroxytyrosol and n-3 fatty acids during pregnancy has no deleterious effects on the reproductive traits of the sows (prolificacy, homogeneity of the litter, and percentage of stillborns and low-birth-weight, LBW, piglets) and the postnatal features of the piglets (growth patterns, adiposity, and metabolic traits). Conversely, in spite of a lower mean weight and corpulence at birth, piglets from the supplemented sows showed higher average daily weight gain and fractional growth rate. Thus, at juvenile stages afterwards, the offspring from the treated group reached higher weight and corpulence, with increased muscle development and better lipidemic and fatty acid profiles, in spite of similar adiposity, than offspring in the control group. However, much caution and more research are still needed before practical recommendation and use in human pregnancies.

## 1. Introduction

There is growing evidence about the benefits of maternal supplementation with antioxidant agents during compromised pregnancies. In this sense, our group has recently shown the positive effect of supplementation with hydroxytyrosol on offspring traits in a swine model of intrauterine growth restriction (IUGR) and low-birth-weight (LBW) [1]). Hydroxytyrosol is a polyphenol present in olive leaves and fruits with even higher antioxidant capacity than vitamin E [2]. Maternal supplementation during pregnancy improves oxidative and metabolic status of IUGR fetuses [3,4] and therefore incidence of LBW neonates and postnatal development of piglets [5,6]. Specifically, hydroxytyrosol diminishes lipid peroxidation and increases the fetal availability of omega-3 and omega-6 polyunsaturated fatty acids (n3 and n6 PUFAs) [4].

The role of fatty acids as metabolic, structural, and signaling molecules makes them especially relevant for favoring fetal development during pregnancy [7]. Fetal availability of fatty acids is strongly dependent on their own synthesis from precursors transferred by the mother. However, some fatty acids, which are indispensable for adequate fetal tissue development and pregnancy success [8], must be obtained from the maternal diet since animals are unable to synthesize them (essential fatty acids, EFAs; [9]). The most important EFAs for fetal development are n3 and n6 PUFAs—specifically, eicosapentaenoic (EPA), docosahexanoic (DHA) and arachidonic (AA) acids [10].

Several studies have now established that maternal dietary intake of n3 and n6 PUFAs plays critical roles during fetal growth and development [11]. Consequently, dietary supplementations during pregnancy, either with α-linolenic acid (ALA, which is a precursor of other PUFAs) or directly with EPA and DHA [12], have increased in popularity [13]. However, systematic benefit-risk analyses and interventional research on PUFAs supplementation during pregnancy have not yet been performed. In this way, the World Health Organization (WHO), although recognizing that n3 PUFAs intake during pregnancy may reduce risk of preterm delivery and occurrence of IUGR and subsequent LBW, states that further research is needed before specific recommendations can be made (https://www.who.int/elena/titles/fish_oil_pregnancy/en/).

Hence, it is necessary to assess the efficacy and safety of dietary supplementations with PUFAs for the development of dietary recommendations and optimal treatment regimens. However, studies in humans are difficult to perform due to ethical issues and confounding factors (genetic and lifestyle heterogeneity as well as the coexistence of co-morbidities and their treatments). Therefore, preclinical investigations in animal models are an essential tool for the systematic analysis of efficacy and safety of new treatments.

There is, moreover, evidence that excessive PUFA intake may reduce the antioxidant capacity of fetuses and newborns, increasing oxidative stress and impairing homeostasis [14,15,16]. Furthermore, n3 PUFAs are prone to oxidation [17], so supplementation of the diet with additional antioxidants is needed. In this sense, concomitant administration of polyphenols like hydroxytyrosol, which also have metabolism-regulatory, anti-inflammatory and immuno-modulatory properties [18], may be useful. In turn, addition of n3 PUFAs may be also positive for hydroxytyrosol-based protocols since hydroxytyrosol raises the fetal availability of n3 and n6 PUFAs but increasing the ratio n6/n3 [4]; increases in the ratio n6/n3 may induce a pro-inflammatory stage and a consequent risk for pregnancy success [11,19].

In view of these considerations, we hypothesize that maternal supplementation with antioxidants and n3 PUFAs would favor fetal development and metabolism, reducing IUGR risks and therefore the postnatal features of the offspring. The present study, using our previously refereed swine model, aimed to test such hypothesis and determine benefits and risks of a dietary supplementation with hydroxytyrosol and n3 PUFAs on developmental and metabolic traits of offspring at IUGR risk.

## 2. Materials and Methods

### 2.1. Ethic Statement

The experiment was assessed and approved by the INIA Committee of Ethics in Animal Research and subsequently by the regional competent authority (report PROEX114/16), according to the Spanish Policy for Animal Protection (RD 53/2013), which meets the European Union Directive 2010/63/UE on the protection of research animals. Sows and piglets were housed at INIA animal facilities, which fulfill the local, national, and European requirements for Scientific Procedures Establishments.

### 2.2. Animals and Experimental Design

The study involved 72 pigs, born from 11 purebred Iberian sows with same age (16 months-old) and similar body-weights (136.7 ± 9.2 kg), which became pregnant after cycle synchronization with altrenogest (Regumate^®^, MSD, Boxmeer, The Netherlands) and insemination with cooled semen from a purebred Iberian boar.

All the sows were fed, from the start of the experimental period until Day 35 of pregnancy, with a standard grain based-food diet with the following mean component values: dry matter, 89.9%; crude protein, 12.3%; fat, 3.6%; metabolizable energy, 2.9 Mcal/kg. Diet analysis showed that the most abundant fatty acids (FA) were palmitic (20.6%), oleic (19.27%) and linolenic acids (41.51%). Food amount was adjusted to fulfill individual daily maintenance requirements based on data from the National Research Council [20].

At Day 35 of pregnancy, all sows were weighed and distributed by body weight in two different groups (control and treated groups). Food amount offered to each sow was restricted to 50% of daily maintenance requirements, which has been previously found to increase the incidence of IUGR [1]. The control group continued being fed with the same diet (group C, *n* = 5), whilst the treated group (group T, *n* = 6) was fed with a diet including 4% of linseed oil and 1.5 mg hydroxytyrosol per kg of feed and the following component values: dry matter: 89.7%; crude protein: 12,4%; fat: 6.2%; and metabolizable energy, 2.9 Mcal/kg. The energy content of diets for groups C and T was balanced by adding sepiolite and other ingredients to the diet offered to the group T. The ingredients of both control and treatment diets are detailed in Table 1, with analysis and fatty acids composition detailed in Supplementary Table S1. After delivery, all the sows in both control and treated groups were fed with the control diet, covering 100% of daily lactation requirements.

At birth, the total number of piglets (both alive and stillborn) was recorded for each sow and sex, weight, and head and body measurements (biparietal diameter, occipito-nasal length, trunk length, and abdominal and thoracic circumferences) were recorded for each piglet. Immediately, all living piglets were tagged for their identification and underwent within-group fostering in order to equalize the number of animals among dams. Piglets remained with sows in individual pens until weaning at 30 days-old, when they were allocated in collective pens after mixing piglets from both treatments for minimizing any effect from pen or batchmates. Piglets were fed with two standards diets adapted to age (30–60 and 60–180 days-old; further data in Supplementary Table S1). The amount of feed offered was re-calculated with age for fulfilling growing requirements.

### 2.3. Assessment of Morphological and Homeostatic Features of Offspring during Early Postnatal Development

All the piglets were again weighted and measured at 15, 30 and 60 days-old. Weight values were used for determining the Average Daily Weight Gain (ADWG) and the Fractional Growth Rate (FGR; weight gained per day per starting weight) for the time-intervals. At 60 days-old, a representative group of 35 piglets (18 controls, 7 females and 11 males, and 17 treated piglets, 8 females and 9 males) were sampled for determining the effects of the maternal supplementation on early-postnatal body-weight and -size, adiposity, body composition, plasma indexes of oxidative stress and antioxidant capacity, plasma parameters of different metabolic pathways and fatty acids composition of subcutaneous fat, muscle, and liver.

### 2.4. Assessment of Morphological and Homeostatic Features of Offspring during Juvenile Development

From 60 to 180 days-old, assessment of growth patterns, adiposity and metabolism was performed monthly in the remaining 37 pigs (17 controls, 7 females and 10 males, and 20 treated pigs, 10 females and 10 males). All the animals were weighted and loin diameter and subcutaneous fat (total back-fat and both inner and outer layers separately) were measured at the P2 point (located at 4 cm from the midline and transversal to the head of the last rib) with a multifrequency linear-array ultrasonographic probe (SV1 Wireless scanner, SonopTek, Beijing, China). The body-weight values were used to determine evolution of ADWG and FGR monthly and during total lifetime. Concomitantly, from the age of 120 days-old onwards, blood samples were monthly drawn from the orbital sinus using sterile 10-mL EDTA vacuum tubes (Vacutainer^®^ Systems Europe, Becton Dickinson, Meylan Cedex, France) after fasting for approximately 16 h. The samples were centrifuged at 1500× *g* for 15 min and the plasma was stored in polypropylene vials at −20 °C until assayed for determination of different plasma metabolic parameters.

### 2.5. Evaluation of Body Composition and Organs Weights of Offspring at 60 and 180 Days-Old

The 37 pigs used for assessment of juvenile development were sampled at 180 days-old, similarly to the sampling performed at 60 days-old, to determine, body composition, plasma indexes of oxidative stress and antioxidant capacity and fatty acids composition of subcutaneous and visceral fat, muscle and liver besides the assessment body-weight and -size, adiposity and plasma metabolic parameters monthly performed.

At both 60 and 180 days-old samplings, pigs were euthanized by stunning and exsanguination in compliance with standard procedures (RD 53/2013). At 60 days-old, a blood sample was drawn using 10-mL EDTA vacuum tubes and processed as previously described. Immediately, body measures (biparietal diameter, occipito-nasal length, trunk length and thoracic and abdominal circumferences) and back-fat depth and loin diameter were recorded as previously described. Afterwards, the head was separated from the trunk at the atlanto-occipital joint and, after recording the ratio of head to body weight, the brain was extracted from the skull and weighed. Then, all thoracic and abdominal viscerae were removed and weighted together. Finally, the major organs (heart, lungs, liver, intestine, kidney, spleen, pancreas, and adrenal glands) were weighed individually for the assessment of possible patterns of asymmetrical IUGR. The following weight ratios were considered: weights of brain, heart, lungs, liver, kidneys, intestine, pancreas, spleen, and adrenal glands relative to total viscera weight.

### 2.6. Evaluation of the Oxidant/Antioxidant Status of the Offspring

Values for total antioxidant capacity were assayed in the plasma samples obtained at 60 and 180 days-old, by using the ferric reducing antioxidant power assay (FRAP) as previously described [21]. Assessment of lipid peroxidation was performed in the same samples by measuring malondialdehyde (MDA) using the thiobarbituric acid reaction [22].

### 2.7. Evaluation of the Metabolic Status of the Offspring

Plasma indexes for metabolism of glucose, lipids and proteins were determined in the plasma samples obtained at 60, 120, 150, and 180 days-old. The glycemic profile was assessed by determining plasma glucose and fructosamine concentrations. Lipids metabolism was assessed by determining plasma concentrations of total cholesterol, high- and low-density lipoproteins cholesterol (HDL-c and LDL-c, respectively), triglycerides and non-esterified fatty acids (NEFA). Protein metabolism was assessed by determining urea and haptoglobin. Metabolic state was also assessed through measuring plasma β-hydroxybutyrate (BHB) and lactate. All metabolites were determined using a clinical chemistry analyzer (Saturno 300 plus, Crony Instruments s.r.l., Rome, Italy), according to manufacturer’s instructions.

### 2.8. Evaluation of Fat Content and Fatty Acid Composition in Feed and Tissue Samples

The fatty acids composition of the feed was determined by extraction and methylation using the protocol described by Sukhija et al. [23]. Fatty acid methyl esters (FAME) were analyzed and identified by gas chromatography (Hewlett Packard HP-6890, Palo Alto, CA, USA) with a flame ionization detector and a capillary column (HP-Innowax, 30 m × 0.32 mm i.d. and 0.25 µm polyethylene glycol-film thickness), with a temperature program of 170 to 245 °C as previously described [24]. Results were expressed as gram per 100 g of detected FAME.

The fat content and fatty acids composition in the pigs were determined in samples of subcutaneous fat, *longissimus dorsi* (LD), *biceps femoris* (BF) and liver, obtained immediately after euthanasia at 60 and 180 days-old; visceral fat was also analyzed at 180 days-old. Intramuscular and liver fat were extracted as described by Segura et al. [25] after lyophilization and homogenization; fat content in each tissue was calculated and expressed as a percentage. The neutral lipid fraction (triglycerides) and the polar lipid fraction (phospholipids) were separated using aminopropyl minicolumns previously activated with 7.5 mL of hexane [26]. Subcutaneous and visceral fat were extracted directly. In the case of back-fat, outer and inner layers were analyzed separately, having in mind that the outer layer is more related to thermoregulation, whereas the inner layer is more metabolically active [27]. The fatty acids composition of all tissues was analyzed using gas chromatography [24]. The quantities of individual fatty acids expressed as g/100 g of total fatty acid content were used to calculate the proportions of saturated fatty acids (SFA), monounsaturated fatty acids (MUFA), polyunsaturated fatty acids (PUFA), and total n3 and n6 FA (∑n3 and ∑n6, respectively), as well as the ratios ∑n6/∑n3 and MUFA/SFA and the unsaturation index (UI; calculated as follows: 1 [% monoenoics] + 2 [% dienoics] + 3 [% trienoics] + 4 [% tetraenoics] + 5 [% pentaenoics] + 6 [% hexaenoics] [28,29]. Furthermore, the desaturation index (DI) was used to determine the activity of the stearoyl-CoA desaturase enzyme 1 (SCD1; ratio of the enzyme product, MUFA mainly oleic acid [C18:1n-9], to the enzyme substrate, SFA mainly stearic acid [C18:0]; [30]). Finally, the activities of the desaturase enzymes for n6 (DN6) and n3 (DN3) were estimated from the ratios C20:4n6/C18:2n6 and C20:5n3/C18:3n3, respectively. ∆9 desaturase activity (D9) was estimated from the ratio (C16:1n7 + C18:1n9)/(C16:1n7 + C18:1n9 + C18:0 + C16:0).

### 2.9. Statistical Analysis

Data were analyzed using SPSS^®^ 25.0 (IBM, Armonk, NY, USA). *t*-student tests were used to assess the effects of independent variables (maternal diet) on the litter size and occurrence of IUGR and subsequent LBW. Piglets with LBW were defined as individuals with a birth-weight of 1 SD below the mean litter birth-weight [31]. Dependent variables related to offspring phenotype (weight, ADWG, back-fat depth, loin diameter, organ weights, indexes of metabolic state and oxidative stress and fatty acids composition) were assessed using two-way ANOVA in a General Linear Model; interactions among potential confounding factors (maternal diet, offspring sex and their interaction) were observed and fixed when statistically significant. Changes over time in weight and measures were assessed by ANOVA for repeated measures with the Green–Houser–Geisser correction when statistically significant. The piglet was the experimental unit. All the results were expressed as mean ± S.E.M. Statistical significance was accepted from *p* < 0.05 and a statistical trend was considered when 0.05 < *p* < 0.1.

## 3. Results

### 3.1. Effects of Maternal Supplementation on Litter Characteristics and Prenatal Development of Offspring

Control and supplemented sows showed similar mean number of total born piglets (8.8 ± 1.8 for group C and 8.8 ± 2.0 for group T; ranging from 6 to 11 piglets in each case), incidence of stillborns (11.36 and 11.32%, respectively) and percentage of LBW neonates (15.9 and 15.4%, respectively). Piglets in the group C showed a higher mean birth-weight and a larger abdominal circumference than treated counterparts (1.3 ± 0.03 vs. 1.2 ± 0.03 kg and 18.7 ± 0.25 vs. 17.8 ± 0.25 cm, respectively; *p* < 0.05 for both). However, litters were similarly homogeneous in both groups, with comparable average birth-weight of the smallest and largest piglets in each litter (0.9 ± 0.1 vs. 0.9 ± 0.1 kg and 1.5 ± 0.2 vs. 1.4 ± 0.2 kg for group C and T, respectively) and within-litter weight ranges (0.6 ± 0.1 vs. 0.5 ± 0.1 kg). Further data are available in Supplementary Table S2.

### 3.2. Effects of Maternal Supplementation on Postnatal Patterns of Growth and Development of Offspring

**Figure 1:**
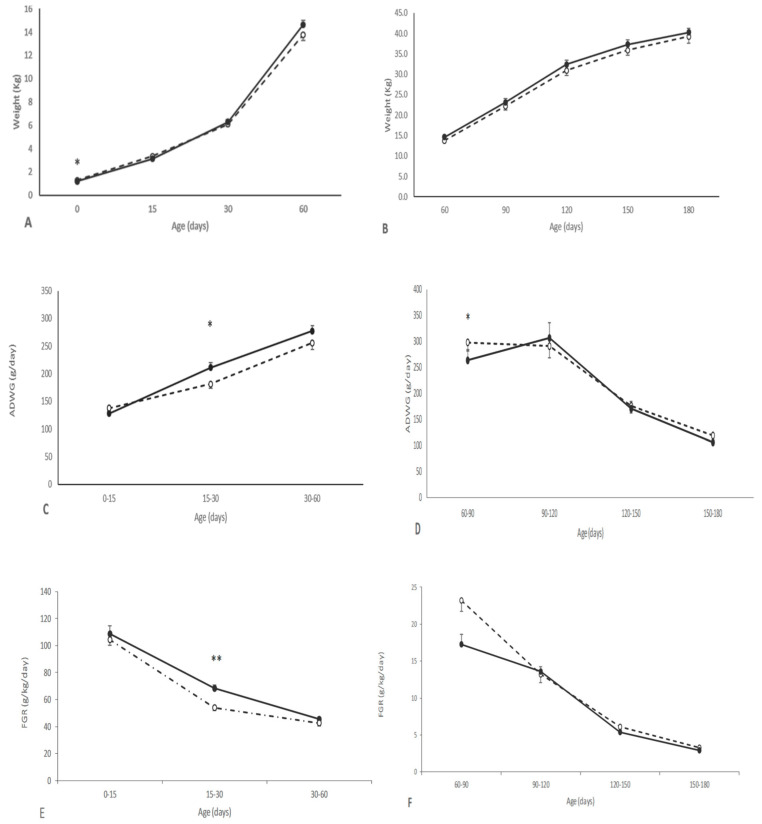
Changes over time in mean values (±S.E.M.) of body weight (**A**–**B**), average daily weight gain (ADWG; panels (**C**,**D**)) and fractional growth rate (FGR; **E**–**F**), during early postnatal (0 to 60 days-old; left panels) and juvenile periods (60 to 180 days-old; right panels), in piglets born from sows treated or not with hydroxytyrosol and linseed oil during pregnancy (groups T (black dots with continuous line) and C (white dots with discontinuous lines), respectively). Asterisks denote significant differences between groups (* *p* < 0.05; ** *p* < 0.01).

The growth of the piglets during the suckling period was positively affected by maternal supplementation during pregnancy. The piglets from the group T showed a higher ADWG and FGR after birth than counterparts in the group C, with the differences being statistically significant between 15 and 30 days-old (*p* < 0.05). Hence, the differences in body weight found at birth were not significant at 15 days and even reverted from 30 to 180 days-old, when pigs in the group T showed higher body-weight values. However, the differences did not reach statistical significance (Figure 1 and Supplementary Tables S2 and S3). At 60 days-old, treated pigs were larger than their control counterparts, showing a larger head size (*p* < 0.05 for both biparietal diameter and occipito-nasal length) and a higher corpulence (*p* < 0.05 for both thoracic and abdominal circumferences). These differences remained over the entire period of study, although without statistical significance at later stages (Supplementary Table S3). There were no significant interactions between treatment and sex.

### 3.3. Effects of Maternal Supplementation on Body Composition and Adiposity of Offspring

There were no significant effects of maternal supplementation on the absolute and relative weights of the different organs (heart, lungs, liver, intestine, kidney, spleen, pancreas and adrenal glands) at both 60 and 180 days-old, excepting absolute and relative weight of the adrenal glands at the age of 180 days (3.7 ± 0.7 vs. 4.1 ± 0.4 g for absolute weight for groups C and T, respectively; *p* < 0.05). The absolute and relative weight of the spleen showed a trend to be heavier in pigs from the group T at 180 days-old (74.1 ± 2.9 vs. 88.0 ± 4.1 g and 0.98 ± 0.03 vs. 1.1 ± 0.05; *p* = 0.60).

**Figure 2:**
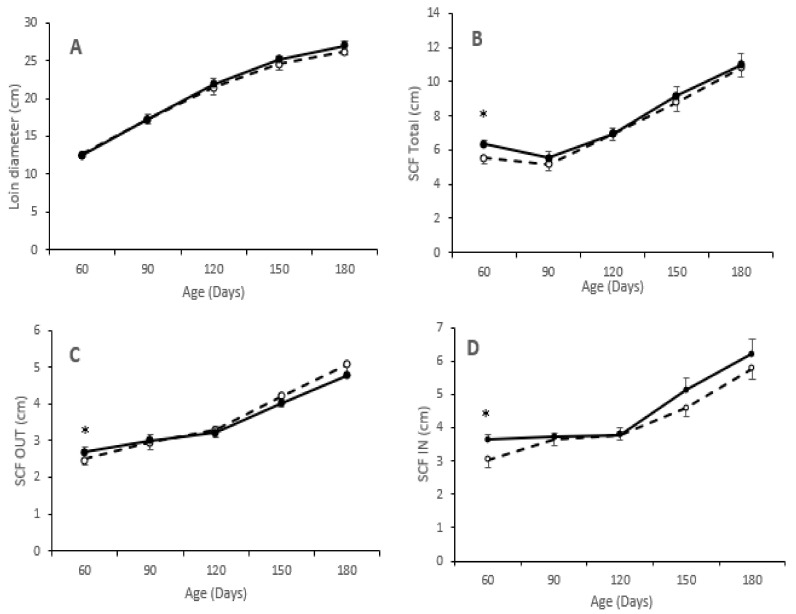
Changes over time in mean values (±S.E.M.) of loin diameter (panel (**A**)) and depth of total subcutaneous back-fat (panel (**B**)) and of its outer and inner layers (panels (**C**,**D**), respectively) during juvenile development (60 to 180 days-old) of pigs born from sows treated or not with hydroxytyrosol and linseed oil during pregnancy (groups T (black dots with continuous line) and C (white dots with discontinuous lines), respectively). Asterisks denote significant differences between groups (* *p* < 0.05).

Assessment of adiposity showed higher values in pigs from the group T than in pigs from the group C at 60 days-old for all measurements of subcutaneous fat depth (*p* < 0.05 for all). However, the values of these variables at the following measurements did not differ between groups, as depicted in Figure 2. Muscle development, measured as loin diameter, was numerically lower from birth to 60 days-old and higher from 60 to 180 days-old in pigs of group T. Assessment of intramuscular fat content in the *longissimus dorsi* showed higher values in pigs from group T, both at 60 (8.5 ± 3.1 vs. 10.2 ± 2.8%: *p* = 0.91) and 180 days-old (8.8 ± 1.7 vs. 12.5 ± 3.7%; *p* < 0.05).

### 3.4. Effects of Maternal Supplementation on the Antioxidant Capacity and Oxidative Stress of Offspring

The values for antioxidant capacity at 60 days-old were significantly affected by the interaction between treatment and sex (Table 2; *p* < 0.05), with males within the T group showing higher FRAP values than their female counterparts (*p* = 0.06). Such effect was not found in control pigs at 60 days-old and in none of the pigs in both groups at 180 days-old. Conversely, malondialdehyde concentrations, at both 60 and 180 days-old, were not affected by maternal supplementation or sex of the pig.

### 3.5. Effects of Maternal Supplementation on the Metabolic Status of Offspring

Maternal supplementation during pregnancy affected the postnatal metabolic status of the pigs (Table 3 and Supplementary Table S4). In brief, the assessment of the plasma glycemic profile showed no significant differences in plasma glucose concentrations. Conversely, lower fructosamine concentrations were found in pigs from treated sows at all ages (*p* < 0.05 at 60 and 120 days-old and *p* = 0.07 at 150 and 180 days-old). The assessment of the lipidemic profile showed that pigs in the group T had lower plasma concentrations of LDL-cholesterol at 60, 120, 150, and 180 days-old (*p* < 0.05 for all) and also lower total and HDL-cholesterol at 120, 150, and 180 days-old (*p* < 0.05 for all). Pigs in the group T also showed statistically significant higher plasma triglycerides concentrations at 60 days-old (*p* < 0.05), but values remained similar from 120 days-old onwards. Finally, NEFA values were always similar between groups excepting higher values in the group T at 120 days-old, concomitantly with lower values of urea (*p* < 0.05 for all). There were no other major differences between groups in the remaining parameters.

### 3.6. Effects of Maternal Supplementation on Fatty Acid Composition of the Offspring

The assessment of fatty acid composition of liver, muscle and fat proved significant differences between pigs from control and treated sows (groups C and T, respectively) at 60 days-old but mainly at 180 days-old (summarized in Table 4 and Table 5, with further data in Supplementary Tables S5–S21).

At 60 days-old, pigs from the group T showed, at both the outer and inner layers of subcutaneous fat, a significant decrease in the ratio of ∑n6/∑n3 FA (*p* < 0.0005 for both layers) and a higher DN6 activity (*p* < 0.005 for both layers). The outer layer in group T also showed a higher content of ∑n3 FA and a lower desaturation index (DI; *p* < 0.05 for both), whereas the inner layer showed higher DN3 activity (*p* < 0.05). The assessment of fatty acid composition in muscle showed main changes at *longissimus dorsi* (LD). Pigs from group T showed, concomitantly with findings at subcutaneous fat, a lower ∑n6/∑n3 ratio at the neutral fraction (*p* < 0.0005). There were higher contents of total PUFA and n6 FA at the polar fraction (*p* < 0.05 for both), and a trend for lower MUFA content (*p =* 0.07) was observed. In the case of the *biceps femoris* (BF), only a lower ∑n6/∑n3 ratio at the neutral fraction and also a higher activity of DN6 at the polar fraction were found (*p* < 0.05). There were fewer changes in the liver, without differences at the neutral fraction and only a higher content of total n3 FA at the polar fraction in the pigs of group T (*p* < 0.05).

At 180 days-old, pigs from groups C and T showed significant differences in fatty acids composition, which were similar among the different compartments. In brief, assessment of the composition of subcutaneous (in both outer and inner layers) and visceral fat in the pigs of the group T showed a higher content of total SFA (*p* < 0.05 for all) and a higher ∑n6/∑n3 ratio (*p* < 0.05 for inner layer and *p* = 0.05 for outer layer of subcutaneous fat, and *p* = 0.09 for visceral fat). There was also a lower content of total n3, lower unsaturation (UI), and DI indexes and lower D9 and DN6 activities (*p* < 0.05 for all) in these pigs. The inner layer of subcutaneous fat also showed lower MUFA/SFA ratio (*p* < 0.005; *p* = 0.08 at the outer layer) and lower PUFA content (*p* < 0.05).

The FA profile of the liver was highly different between groups, mainly at the polar fraction. Changes were similar to those found at subcutaneous and visceral fat. Hence, pigs from the group T showed a higher content of SFA (*p* < 0.05), a lower content of MUFA and PUFA (*p* < 0.05 for all) and, therefore, lower MUFA/SFA ratio (*p* < 0.01). In the polar fraction of the liver, we also observed a lower content of total n3 and n6 FA in the T group (*p* < 0.0005 and *p* < 0.05, respectively) and a trend for a higher n6/n3 ratio (*p =* 0.08). The group T also showed lower UI and DI indexes and D9 activity than the group C (*p* < 0.0005, *p* < 0.05 and *p* < 0.01, respectively). The assessment of the neutral fraction of the liver in pigs from group T showed lower contents of PUFA and total n6 FA with lower activity of DN6 (*p* < 0.05 for all) and higher DI (*p* < 0.005) than pigs from group C.

Finally, the assessment of the LD muscle showed no changes at the polar fraction but a trend for lower PUFA content (*p =* 0.05) and significantly lower ∑n6 content, ∑n6/∑n3 ratio and UI (*p* < 0.05 for all) in the group T. Conversely, evaluation of fatty acids at BF showed no differences at the neutral fraction and only a higher DI at the polar fraction of pigs in group T (*p* < 0.05).

## 4. Discussion

The results obtained in the present study indicate that maternal supplementation with hydroxytyrosol and omega-3 fatty acids during pregnancy has no deleterious effects on the reproductive traits of the sows (prolificacy, homogeneity of the litter, and percentage of stillborns and LBW piglets) and the postnatal features of the piglets (growth patterns, adiposity, and metabolic traits). Conversely, in spite of a lower mean weight and corpulence at birth, piglets from the supplemented sows showed afterwards higher average daily weight gain (ADWG) and fractional growth rate (FGR). Thus, at juvenile stages, the offspring from the treated group reached higher weight and corpulence, with increased muscle development and better lipidemic and fatty acids profiles, in spite of similar adiposity, than offspring in the control group.

The assessment of birth-weight showed that the neonates from treated sows had a mean value around 8% lower than the piglets from control dams, although such lower birth-weight was not accompanied by a higher incidence of LBW piglets or a higher heterogeneity of the litter. In fact, mean birth-weight of smallest and largest piglets and the range of within-litter weights were similar between controls and treated litters. The decrease in mean birth-weight found in the present study differs from the results obtained after maternal supplementation with only hydroxytyrosol [5]. In such study, mean birth-weight was increased whilst the incidence of LBW piglets was decreased. At a first glance, these differences between studies may be related to the incorporation of n3 fatty acids. However, the negative effect of n3 fatty acids on birth-weight has not been reported to the date, so further research would be necessary to clarify such findings.

In fact, despite a positive relationship between maternal n3 fatty acids availability and birth outcomes has been described [32], the information on the effects of n3 supplementation during the pregnancy of different species is not conclusive. Some studies in rats [33], rabbits [34], and humans [35] have shown positive effects on birth-weight. On the contrary, other studies in human pregnancies indicate that n3 supplementation would be more related to the prevention of preterm births than to reduction of LBW incidence [36]. Prolongation of gestation, by a lesser susceptibility to preterm parturition, would increase the size and weight of newborns in pregnancies at risk of preterm delivery. The same pattern has been described in sheep models [37,38], and in some studies in pig models when they were given fish oil [39,40]. However, we didn’t find evidence in the current study. In the case of pigs, Smit and co-workers [41] reported no effects of maternal n3 intake on birth-weight, but a lower number of total and alive newborns. These results, which are opposite to our present findings, reinforce the need for further studies to elucidate the effects of maternal supplementation with n3 fatty acids on birth outcomes.

The assessment of piglets’ features during early postnatal stages (lactation and early post-weaning) indicated, firstly, similar growth patterns (in terms of both ADWG and FGR) between offspring from control and treated sows from birth to 15 days-old. Afterwards, at the second half of lactation, both ADWG and FGR changed to be significantly higher in piglets from treated sows. Hence, these piglets reversed differences in birth-weight for reaching a higher body-weight and -size than their control counterparts at 60 days-old; piglets from treated sows also showed improved muscle and fat accretion. These results prove *catch-up* growth during the second half of lactation and, afterwards, during early post-weaning. In fact, it is known that post-weaning development is strongly dependent on the growth achieved during lactation and therefore on the body weight reached at weaning [42].

These positive effects of maternal supplementation with n3 fatty acids on early postnatal development were accompanied by significant changes in the fatty acid composition of both subcutaneous and intramuscular fat. At 60 days-old, pigs from treated sows showed, at the outer and inner layers of subcutaneous fat and at the neutral fraction of the intramuscular fat (composed all of them by triglycerides), a significant decrease in the ratio of n6/n3 FA. This is an interesting finding because it may be protective against possible metabolic disorders by diminishing pro-inflammatory status and favoring the action of insulin [43]. The higher content of n3 FA and the lower desaturation index found at the outer layer also indicate a protective metabolic status. Firstly, a higher availability of n3, which was also found at the polar fraction of the liver, improves pro-/anti-inflammatory status and reduces pathological risks due to a higher availability of anti-inflammatory lipids mediators [44,45]. Conversely, a higher availability of n3 also improves insulin function and physical and mental development during early postnatal stages [46,47,48]. Concomitantly, a lower desaturation index is indicative of adequate insulin sensitivity [30,49,50,51].

The cause for these significant changes in the availability of n3 and the ratio ∑n6/∑n3 at early postnatal stages is, however, a point that needs to be elucidated through further research. Our pigs, like other mammals, cannot synthesize essential fatty acids and have to acquire them from the diet. However, the postnatal diet was the same in the group C and T and the maternal supplementation was stopped at birth, two months prior to sampling the pigs. Hence, in the absence of different diet supply of essential fatty acids, we can hypothesize differences on bioavailability, by increased amount or speed of absorption and/or differences in the desaturase or elongase pathways [17,52]. Such hypothesis may be supported by the data of present experiment evidencing that both outer and inner layers of subcutaneous fat and the polar fraction of liver showed higher DN6 activity. Moreover, the inner layer (more metabolically active [27]) also showed higher DN3 activity. However, further and specifically focused research need to be undertaken to elucidate additional mechanisms.

The assessment of piglets’ features during late postnatal stages (juvenile development) showed higher values of phenotypic characteristics (body-weight, back-fat depth and loin diameter) in the pigs from treated sows, in agreement with previous studies on swine developmental patterns [53,54,55] and, specifically, in studies focused on maternal supplementation with only hydroxytyrosol [5,6] or only n3 fatty acids [41,56].

Similar to earlier stages, significant differences in the fatty acids composition of pigs from control and treated sows were also found at 180 days-old. There were significant differences with the fatty acids profile found at 60 days-old, which indicates that changes are biphasic and depending on age. These changes were very consistent among subcutaneous (in both outer and inner layers) and visceral fat. These changes, in brief, consisted of a higher content of total SFA and a higher ∑n6/∑n3 FA ratio associated with a lower content of total n3 FA, lower unsaturation and desaturation indexes, and lower D9 and DN6 activities. The inner layer of subcutaneous fat, more metabolically active as said previously [27], showed a lower MUFA/SFA ratio and a lower total PUFA content. All these changes were found exactly at the polar fraction of the liver, which reinforces their consistency. Such fraction also evidenced a lower content of total MUFA and n6 FA. The neutral fraction of the liver also showed lower content of PUFA, associated with lower total n6 FA, lower activity of DN6, and a higher desaturation index. The increase of ∑n6/∑n3 ratio with lower ∑n3 content indicates a pro-inflammatory state related to increased peripheral lipolysis and, therefore, an enlarged flux of fatty acids [57]. Concomitantly, a decreased PUFA content (specifically n6 PUFA) is related to altered states of fat accretion and lipids metabolism with the MUFA content, and the MUFA/SFA ratio and unsaturation and desaturation indexes increased [49,50,51,57]. However, this is not the case of the present study.

In fact, pigs from treated sows showed better metabolic phenotype during early development. The glycemic profile showed did not differ significantly in terms of plasma glucose concentrations but lower values for fructosamine in pigs from treated sows were observed. Fructosamine is indicative of precedent glucose availability, so such data may indicate that the maternal supplementation would protect from insulin resistance despite a higher compensatory growth and adiposity. In the same way, the assessment of the lipidemic profile showed that pigs from treated sows also had a protective lipids phenotype, supporting previous data in rats [58] and humans [59], with lower plasma concentrations of total and LDL-cholesterol from 120 days-old onwards. We have to note that pigs from treated sows in our study showed higher plasma triglycerides concentrations at 60 days-old, but we can hypothesize that this is a transitory effect of the higher adiposity reached during early development. Hence, the results of the present study seem to indicate that maternal supplementation with n3 FA and hydroxytyrosol is beneficial for the metabolic status and health of their offspring. However, the increased ∑n6/∑n3 ratio with lower n3 and higher n6 content gives a warning about the need for more specific research prior to recommend the use of such supplementation.

## 5. Conclusions

The results obtained in the present study indicate that the maternal supplementation with hydroxytyrosol and omega-3 fatty acids during pregnancy appears to be effective, with no major associated complications, for improving postnatal growth patterns, metabolic traits, and carcass and meat quality during juvenile development. Previous studies credited the amenability of the Iberian pig for translational research in human nutrition and metabolism [1,60,61,62,63]. However, despite the beneficial effects found in the current study and having in mind some changes in fatty acids profile, much caution and more research are still needed before practical recommendation and use, especially in human pregnancies.

## Figures and Tables

**Table 1:** Ingredient composition of experimental diets of sows (C = control group; T = treated group).

Ingredients (g/Kg)	Group	
	C	T
Barley	234.0	299.0
Wheat	300.1	192.5
Wheat bran	245.5	300.0
Cookie Flour	100.0	0.0
Beet pulp	50.0	50.0
Sunflower flour (28%)	31.5	68.0
Soybean oil	7.0	0.0
Linseed oil	0.0	40.0
Sepiolite	0.0	18.1
Salt	5.0	5.0
Phosfate Monocalcium	2.5	2.5
Calcium carbonate	16.5	17.5
L—Lysine (500g/kg)	4.2	3.8
L-Threonine	0.7	0.6
Mineral and vitamin Premix	3.0	3.0

**Table 2:** Mean plasma concentrations (± S.E.M.) for total ferric antioxidant capacity (FRAP) and lipid peroxidation (malondialdehyde; MDA) in pigs born from sows treated or not with hydroxytyrosol and linseed oil during pregnancy (groups T and C, respectively) at 60 and 180 days-old.

Days	Parameter	C			T		
		Mean	Female	Male	Mean	Female	Male
60	Number of piglets	18	7	11	17	8	9
	FRAP (µmol/mL)	18.5 ± 1.18	17.2 ± 0.49	19.3 ± 1.91	21.3 ± 2.01	17.4 ^a^ ± 2.00	24.8 ^b^ ± 2.98
	MDA (µmol/L)	75.3 ± 5.43	81.4 ± 7.20	71.4 ± 7.65	65.4 ± 3.09	70.7 ± 4.66	60.8 ± 3.65
180	Number of piglets	17	7	10	20	10	10
	FRAP (µmol/mL)	38.1 ± 3.33	37.9 ± 7.18	38.3 ± 3.53	42.6 ± 4.74	49.2 ± 6.61	36.8 ± 6.61
	MDA (µmol/L)	27.2 ± 2.41	27.5 ± 4.76	27.1 ± 2.63	29.6 ± 1.96	31.4 ± 3.02	27.6 ± 2.42

Different superscripts indicate a tendency of significant differences a ≠ b; *p* = 0.06.

**Table 3:** Changes over time in mean plasma concentrations (±S.E.M.) for main metabolic parameters in pigs born from sows treated or not with hydroxytyrosol and linseed oil during pregnancy (groups T, *n* = 20, and C, *n* = 17, respectively).

Metabolites	60 Days		120 Days		150 Days		180 Days	
	C	T	C	T	C	T	C	T
GLU (MG/DL)	100 ± 4.95	99.0 ± 5.63	97.4 ± 2.64	92.1 ± 2.26	105 ± 4.14	97.3 ± 2.12	99.3 ± 2.87	99.4 ± 3.31
FRU (MG/DL)	309 ^a^ ± 14.5	254 ^b^ ± 16.4	250 ^c^ ± 4.71	231 ^d^ ± 6.17	234 ± 5.47	225 ± 4.95	235 ± 4.84	222 ± 5.04
CHO (MG/DL)	107 ± 5.41	96.0 ± 3.79	111 ^a^ ± 2.85	101 ^b^ ± 1.88	109 ^c^ ± 2.33	99.5 ^d^ ± 2.30	106 ^e^ ± 2.98	97.0 ^f^ ± 3.13
HDL (MG/DL)	31.1 ± 1.45	28.8 ± 1.44	42.4 ^a^ ± 0.95	39.3 ^b^ ± 0.68	43.8 ^c^ ± 1.40	39.0 ^d^ ± 0.89	38.2 ^e^ ± 1.13	35.3 ^f^ ± 1.11
LDL (MG/DL)	78.2 ^a^ ± 5.10	63.2 ^b^ ± 2.98	66.4 ^c^ ± 2.60	59.7 ^d^ ± 1.97	64.7 ^e^ ± 3.01	57.2 ^f^ ± 1.96	73.2 ^g^ ± 3.23	64.8 ^h^ ± 2.47
TG (MG/DL)	50.3 ^a^ ± 5.81	69.8 ^b^ ± 4.74	63.8 ± 4.89	59.9 ± 4.14	52.4 ± 2.55	46.7 ± 2.77	46.3 ± 2.83	49.9 ± 3.51
NEFA (MMOL/L)	0.42 ± 0.02	0.43 ± 0.04	0.49 ^a^ ± 0.02	0.51 ^b^ ± 0.02	0.37 ± 0.02	0.38 ± 0.02	0.57 ± 0.05	0.52 ± 0.04
UREA (MG/DL)	25.3 ± 1.77	24.4 ± 1.58	26.3 ^a^ ± 1.92	21.6 ^b^ ± 0.99	29.1 ± 1.86	28.8 ± 2.17	29.0 ± 2.42	27.9 ± 3.14
HP (MG/DL)	55.6 ± 3.25	46.8 ± 4.74	37.7 ± 2.69	35.8 ± 2.44	42.9 ± 4.56	47.2 ± 3.77	44.4 ± 3.69	51.0 ± 4.20
LAC (MG/DL)	48.8 ± 5.15	44.6 ± 5.55	30.1 ± 2.64	21.6 ± 3.61	44.3 ± 6.95	36.9 ± 4.79	52.6 ± 4.74	49.0 ± 4.62
BHB (MMOL/L)	0.35 ± 0.03	0.32 ± 0.04	0.20 ± 0.02	0.25 ± 0.04	0.32 ± 0.03	0.30 ± 0.03	0.32 ± 0.03	0.29 ± 0.03

GLU = glucose; FRU = fructosamine; CHO = cholesterol; HDL = high density lipoprotein cholesterol; LDL = low density lipoprotein cholesterol; TG = triglycerides; NEFA = non-esterified fatty acids; HP = haptoglobin; LAC = lactate; BHB = B-hydroxybutyrate. Different superscripts indicate significant differences (a ≠ b; c ≠ d; e ≠ f; g ≠ h; all *p* < 0.05).

**Table 4:** Highlight of significant differences (*p* < 0.05) ± S.E.M. in fatty acid composition, at 60 days-old, between pigs born from sows treated or not with hydroxytyrosol and linseed oil during pregnancy (groups T, *n* = 17, and C, *n* = 18, respectively). Complete data are offered in Supplementary Tables S5–S12.

Tissue	Fraction	Variable	C	T
SCF	Out	∑n3 (g/100g FA)	2.09 ^a^ ± 0.05	2.24 ^b^ ± 0.06
		∑n6/∑n3	10.6 ^e^ ± 0.11	10.0 ^f^ ± 0.09
		DN6	0.007 ^c^ ± 0.00	0.009 ^d^ ± 0.000
		DI	6.55 ^a^ ± 0.33	5.66 ^b^ ± 0.18
	In	∑n6/∑n3	10.8 ^e^ ± 0.14	10.0 ^f^ ± 0.13
		DN3	0.021 ^a^ ± 0.000	0.022 ^b^ ± 0.000
		DN6	0.007 ^e^ ± 0.000	0.008 ^f^ ± 0.000
LD	Neutral	∑n6/∑n3	9.86 ^e^ ± 0.11	9.18 ^f^ ± 0.10
	Polar	PUFA (g/100g FA)	43.7 ^a^ ± 0.38	45.2 ^b^ ± 0.45
		∑n6	40.5 ^a^ ± 0.37	41.8 ^b^ ± 0.45
BF	Neutral	∑n6/∑n3	10.6 ^a^ ± 0.19	9.78 ^b^ ± 0.15
	Polar	DN6	0.32 ^a^ ± 0.04	0.35 ^b^ ± 0.04
Liver	Polar	∑n3 (g/100g FA)	2.14 ^a^ ± 0.16	2.55 ^b^ ± 0.09

SCF = subcutaneous fat; LD = *longissimus dorsi*; BF = *biceps femoris*; FA = fatty acids; DN6 = total activity for the desaturases enzymes of n6; DN3 = total activity for the desaturases of n3; DI = desaturation index; PUFA = sum of polyunsaturated FA; MUFA = sum of monounsaturated FA. Different superscripts indicate significant differences a ≠ b (*p* < 0.05); c ≠ d (*p* < 0.01); e ≠ f (*p* < 0.0005).

**Table 5:** Highlight of significant differences (*p* < 0.05) ± S.E.M. in fatty acid composition, at 180 days-old, between pigs born from sows treated or not with hydroxytyrosol and linseed oil during pregnancy (groups T, *n* = 20, and C, *n* = 17, respectively). Complete data are offered in Supplementary Tables S13–S21.

Tissue	Fraction	Variable	C	T
SCF	Out	∑n3 UI DI	1.19 ^a^ ± 0.03 0.88 ^a^ ± 0.01 5.11 ^c^ ± 0.11	1.10 ^b^ ± 0.03 0.86 ^b^ ± 0.01 4.64 ^d^ ± 0.10
		D9	0.61 ^c^ ± 0.003	0.60 ^d^ ± 0.004
		DN6	0.016 ^a^ ± 0.00	0.013 ^b^ ± 0.00
	In	SFA PUFA MUFA/SFA	34.3 ^c^ ± 0.41 15.7 ^a^ ± 0.54 1.41 ^c^ ± 0.02	35.9 ^d^ ± 0.34 14.6 ^b^ ± 0.36 1.34 ^d^ ± 0.02
		∑n6/∑n3	13.7 ^a^ ± 0.20	15.1 ^b^ ± 0.31
		∑n3 UI DI	1.07 ^c^ ± 0.04 0.84 ^c^ ± 0.01 3.96 ^c^ ± 0.08	0.92 ^d^ ± 0.03 0.81 ^d^ ± 0.01 3.55 ^d^ ± 0.07
VCF		∑n3 UI DI	0.95 ^a^ ± 0.03 0.73 ^a^ ± 0.01 2.11 ^a^ ± 0.05	0.91 ^b^ ± 0.03 0.72 ^b^ ± 0.01 1.99 ^b^ ± 0.03
LD	Neutral	∑n6 ∑n6/∑n3 UI	9.48 ^a^ ± 0.49 10.75 ^a^ ± 0.29 0.77 ^a^ ± 0.01	8.17 ^b^ ± 0.41 9.96 ^b^ ± 0.20 0.74 ^b^ ± 0.01
BF	Polar	DI	1.12 ^a^ ± 0.03	1.20 ^b^ ± 0.05
Liver	Neutral	PUFA ∑n6 DN6	8.96 ^a^ ± 0.92 7.24 ^a^ ± 0.85 0.38 ^c^ ± 0.01	8.67 ^b^ ± 0.50 6.61 ^b^ ± 0.46 0.34 ^d^ ± 0.01
	Polar	SFA MUFA PUFA MUFA/SFA ∑n3 ∑n6 UI DI D9	62.0 ^a^ ± 0.64 23.2 ^a^ ± 0.50 14.9 ^c^ ± 0.67 0.37 ^c^ ± 0.01 1.68 ^e^ ± 0.08 13.2 ^a^ ± 0.60 0.69 ^e^ ± 0.02 0.63 ^a^ ± 0.02 0.25 ^c^ ± 0.01	63.8 ^b^ ± 0.42 21.7 ^b^ ± 0.32 14.5 ^d^ ± 0.44 0.34 ^d^ ± 0.01 1.55 ^f^ ± 0.04 12.9 ^b^ ± 0.41 0.65 ^f^ ± 0.02 0.56 ^b^ ± 0.01 0.23 ^d^ ± 0.00

SCF = subcutaneous fat; VCF = visceral fat; LD = *longissimus dorsi*; BF = *biceps femoris*; FA = fatty acids; DN6 = total activity for the desaturases enzymes of n6; DN3 = total activity for the desaturases of n3; DI = desaturation index; PUFA= sum of polyunsaturated FA; MUFA = sum of monounsaturated FA; SFA = sum of saturated fatty acids; UI = unsaturation index; DI = desaturation index; D9 = activity of ∆9 desaturase. Different superscripts indicate significant differences a ≠ b (*p* < 0.05); c ≠ d (*p* < 0.01); e ≠ f (*p* < 0.0005).

## Supplementary Materials

The following are available online at https://www.mdpi.com/2076-2615/10/11/1946/s1, Table S1: Analysis (g/kg, dry-matter basis) and fatty acid composition of diets, Table S2: Body weight (mean Kg ± S.E.M.) in piglets born from sows treated or not with hydroxytyrosol and linseed oil during pregnancy (groups T and C, respectively) at 0, 15, 30, 60, 90, 120, 150 and 180 days-old, Table S3: Body measures (mean ± S.E.M.) in piglets born from sows treated or not with hydroxytyrosol and linseed oil during pregnancy (groups T and C, respectively) at 0, 15, 30, 60 and 180 days-old, Table S4: Changes over time in mean plasma concentrations (± S.E.M.) for main metabolic parameters in piglets born from sows treated or not with hydroxytyrosol and linseed oil during pregnancy (groups T and C, respectively), Table S5: Differences in fatty acid composition (g/kg ± S.E.M.) and desaturase activity in outer layer of the subcutaneous fat between piglets born from sows treated or not with hydroxytyrosol and linseed oil during pregnancy (groups T and C, respectively) at 60 days-old, Table S6: Differences in fatty acid composition (g/kg ± S.E.M.) and desaturase activity in the inner layer of the subcutaneous fat between piglets born from sows treated or not with hydroxytyrosol and linseed oil during pregnancy (groups T and C, respectively) at 60 days-old, Table S7: Differences in fatty acid composition (g/kg ± S.E.M.) and desaturase activity in the neutral fraction of the longissimus dorsi muscle between piglets born from sows treated or not with hydroxytyrosol and linseed oil during pregnancy (groups T and C, respectively) at 60 days-old, Table S8: Differences in fatty acid composition (g/kg ± S.E.M.) and desaturase activity in the polar fraction of the longissimus dorsi muscle between piglets born from sows treated or not with hydroxytyrosol and linseed oil during pregnancy (groups T and C, respectively) at 60 days-old, Table S9: Differences in fatty acid composition (g/kg ± S.E.M.) and desaturase activity in the neutral fraction of the biceps femoris muscle between piglets born from sows treated or not with hydroxytyrosol and linseed oil during pregnancy (groups T and C, respectively) at 60 days-old, Table S10: Differences in fatty acid composition (g/kg ± S.E.M.) and desaturase activity in the polar fraction of the biceps femoris muscle between piglets born from sows treated or not with hydroxytyrosol and linseed oil during pregnancy (groups T and C, respectively) at 60 days-old, Table S11: Differences in fatty acid composition (g/kg ± S.E.M.) and desaturase activity in the neutral fraction of the liver between piglets born from sows treated or not with hydroxytyrosol and linseed oil during pregnancy (groups T and C, respectively) at 60 days-old, Table S12: Differences in fatty acid composition (g/kg ± S.E.M.) and desaturase activity in the polar fraction of the liver between piglets born from sows treated or not with hydroxytyrosol and linseed oil during pregnancy (groups T and C, respectively) at 60 days-old, Table S13: Differences in fatty acid composition (g/kg ± S.E.M.) and desaturase activity of the visceral fat piglets born from sows treated or not with hydroxytyrosol and linseed oil during pregnancy (groups T and C, respectively) at 180 days-old, Table S14: Differences in fatty acid composition (g/kg ± S.E.M.) and desaturase activity in the outer layer of the subcutaneous fat piglets born from sows treated or not with hydroxytyrosol and linseed oil during pregnancy (groups T and C, respectively) at 180 days-old, Table S15: Differences in fatty acid composition (g/kg ± S.E.M.) and desaturase activity in the inner layer of the subcutaneous fat piglets born from sows treated or not with hydroxytyrosol and linseed oil during pregnancy (groups T and C, respectively) at 180 days-old, Table S16: Differences in fatty acid composition (g/kg ± S.E.M.) and desaturase activity in the neutral fraction of the longissimus dorsi muscle between piglets born from sows treated or not with hydroxytyrosol and linseed oil during pregnancy (groups T and C, respectively) at 180 days-old, Table S17: Differences in fatty acid composition (g/kg ± S.E.M.) and desaturase activity in the polar fraction of the longissimus dorsi muscle between piglets born from sows treated or not with hydroxytyrosol and linseed oil during pregnancy (groups T and C, respectively) at 180 days-old, Table S18: Differences in fatty acid composition (g/kg ± S.E.M.) and desaturase activity in the neutral fraction of the biceps femoris muscle between piglets born from sows treated or not with hydroxytyrosol and linseed oil during pregnancy (groups T and C, respectively) at 180 days-old, Table S19: Differences in fatty acid composition (g/kg ± S.E.M.) and desaturase activity in the polar fraction of the biceps femoris muscle between piglets born from sows treated or not with hydroxytyrosol and linseed oil during pregnancy (groups T and C, respectively) at 180 days-old, Table S20: Differences in fatty acid composition (g/kg ± S.E.M.) and desaturase activity in the neutral fraction of the liver between piglets born from sows treated or not with hydroxytyrosol and linseed oil during pregnancy (groups T and C, respectively) at 180 days-old, Table S21: Differences in fatty acid composition (g/kg ± S.E.M.) and desaturase activity in the polar fraction of the liver between piglets born from sows treated or not with hydroxytyrosol and linseed oil during pregnancy (groups T and C, respectively) at 180 days-old.
